# Benefits of Grandparental Caregiving in Chinese Older Adults: Reduced Lonely Dissatisfaction as a Mediator

**DOI:** 10.3389/fpsyg.2020.01719

**Published:** 2020-07-24

**Authors:** Yuanqing Chang, Yin Li, Xin Zhang

**Affiliations:** ^1^School of Psychological and Cognitive Sciences, Peking University, Beijing, China; ^2^Beijing Key Laboratory of Behavior and Mental Health, Peking University, Beijing, China

**Keywords:** intergenerational care, physical and mental health, lonely dissatisfaction, cognitive functions, longitudinal follow-up

## Abstract

**Objective:**

The purpose of the present study is twofold: (1) to investigate the differences in terms of physical and mental health between those who provide grandparental care and those who do not and (2) to explore the mechanism that connects grandparental caregiving and health-related outcomes.

**Methods:**

Two studies (a cross-sectional and a short-term longitudinal follow-up) were conducted. The cross-sectional study (Study 1) examined 148 older adults who provided grandparental care and another 150 older adults who did not. A small longitudinal follow-up study (Study 2) was conducted among 102 older adults randomly selected from Study 1, of which 52 were older adults who provided grandparental care, and another 50 older adults were those who did not. Health status (measured by SF-36), lonely dissatisfaction (measured by Lonely Dissatisfaction Subscale of PGC-MS), and cognitive functions (measured by subscales of WAIS) as well as demographics were measured in both studies.

**Results:**

Results of both the cross-sectional and longitudinal studies showed that, compared with older adults who did not provide grandparental care, those providing grandparental care had significantly better physical and mental health as well as reduced lonely dissatisfaction. Further path analysis showed that lonely dissatisfaction mediated the association between providing grandparental care and enhancement in functions such that providing grandparental care could reduce lonely dissatisfaction, which, in turn, could improve their physical and mental health even after controlling for their cognitive functions.

**Discussion:**

These results suggest that providing grandparental care can improve older adults’ physical and mental health through reduced lonely dissatisfaction.

## Introduction

Due to increased life expectancy, older adults now have more years to spend with grandchildren (e.g., providing grandparental care) than ever before in China and worldwide. Data from the China Health and Retirement Longitudinal Study (CHARLS) revealed that, in 2015, nearly 53% of the sampled middle-age or older adults would provide grandparental care to at least one grandchild under the age of 16 (Wu, 2018). However, the effect of grandparental caregiving on grandparents is controversial. For example, some research reports negative health effects of caring for grandchildren (Minkler et al., 1997; Szinovacz et al., 1999; Minkler and Fuller-Thomson, 2001; Blustein et al., 2004; Lo and Liu, 2009; Lou, 2011; Chen and Liu, 2012). On the contrary, other studies find health benefits for grandparents who provide care to grandchildren, such as reduced depressive symptoms (Silverstein et al., 2006; Cong and Silverstein, 2008; Tsai et al., 2013), better self-rated health (Ku et al., 2013), greater life satisfaction (Silverstein et al., 2006; Grundy et al., 2012; Xu et al., 2012), and even improved cognition (Arpino and Bordone, 2014; Burn and Szoeke, 2015; Ahn and Choi, 2019; Sneed and Schulz, 2019). Moreover, the underlying mechanism of how grandparental caregiving affects caregivers’ health has yet to be identified and described. Hence, the present study investigated the effect of grandparental caregiving on grandparents’ physical and mental health with cross-sectional and longitudinal data, aiming at filling the research gap. We would also like to further explore the role of lonely dissatisfaction in mediating the association between grandparental caregiving and the associated outcomes.

## Grandparental Caregiving in China

In the literature, grandparental caregiving is defined as grandparents taking care of their grandchildren either alone or as a parent’s assistant (Fuller-Thomson et al., 1997; Hirshorn, 1998; Hayslip and Kaminski, 2005) and two forms of grandparental caregiving could be clustered – i.e., (1) custodial grandparental caregiving, a form in which parents might be missing due to substance abuse, marital breakdown, child abuse, incarceration of adult child, or parents’ migration to work in overseas and in cities and grandparents are the sole or primary caregivers (Jendrek, 1994; Fuller-Thomson et al., 1997; Hirshorn et al., 2000; Goodman and Silverstein, 2002; Silverstein et al., 2006) and (2) co-parenting, which, in contrast to custodial grandparental caregiving, involves multigenerational families (Hermalin et al., 1998; Silverstein et al., 2006; Chen and Liu, 2012; Ku et al., 2013) wherein both generations (i.e., parents and grandparents) take responsibility for caring for the child(ren) in the family. In the present study, we are referring to the latter form as it is the major type in China and research has reported that the proportion of grandparents living with and taking care of grandchildren ages 0–6 reached 45% or more (e.g., Chen et al., 2011).

Rooted in the Confucian cultural tradition of familism, taking care of grandchildren is the most salient behavior of Chinese grandparents (Strom et al., 1999; Chen and Liu, 2012). Chinese grandparents provide childcare not only as a result of cultural expectation but also to provide instrumental support to their offspring. In recent years, due to the growth of industrialism and urbanization, the family structure in China has changed dramatically. For instance, the increasing rise of migrant nuclear households in large cities has required many grandparents to move to cities to provide care for their grandchildren (Zeng and Wang, 2018). According to the 2016 Report on China’s Migrant Population Development, providing grandparental care for grandchildren has become the main reason for older adults deciding to migrate to major cities. On average, about 43% of older adults who migrated to major cities were providing grandparental care for their grandchildren, and in big cities like Guangzhou and Shenzhen, the numbers increased to 51.7 and 53.2%, respectively. Such older adults were also labeled as migrant grandparents, defined as grandparents who move to cities to provide grandparental care for their migrant adult children (Arber and Timonen, 2012; King et al., 2014; Qi, 2018).

Grandparental caregiving in migrant households has shown distinct characteristics different from traditional multigenerational household grandparental caregiving, which could influence the health effects of grandparental caregiving. In migrant households, grandparents do not necessarily live with their adult children for a long time, and grandparents moved to the city where their adult children lived mainly to provide short-term or period grandparental caregiving. When the grandchild was old enough to take care of themselves or during other occasions (such as public holidays), grandparents would go back to their hometown. For example, an analysis conducted by Yasuda and colleagues revealed that adult children who moved to a large city from a smaller city or rural area were less likely to live with their older parents (Yasuda et al., 2011).

Findings drawing on the associations between multigenerational grandparental caregiving and health consequences in China are still sparse and inconclusive, which could be largely attributed to socioeconomic disparities between rural and urban areas (Silverstein et al., 2006; Ku et al., 2013; Choi, 2019). For example, research from rural mainland China, Taiwan, and Hong Kong found that grandparents who provided multigenerational household grandparental care reported fewer depressive symptoms and greater life satisfaction relative to non-caregiving grandparents (Silverstein et al., 2006; Ku et al., 2013). However, Choi (2019) further distinguished differential associations between grandparental caregiving and depressive symptoms for rural and urban Chinese grandparents, and the results show that, in rural China, compared to non-caregiving counterparts, the depressive symptoms of grandparents who provided multigenerational household grandparental caregiving increased with age although, for urban grandparental caregiving, there were no significant associations between grandparental care and depression. In addition, up to now, to our best knowledge, the health implications of migrant multigenerational household grandparental caregiving in urban China has yet to be explored. Hence, given the rapid urbanization growth and the distinct nature of migrant multigenerational grandparental care, our present study mainly focuses on the health consequences of migrant household grandparental caregiving in the urbanization context in China.

## Grandparental Caregiving Boosts Social Connectedness

In the case of migrant grandparents, it has been argued that grandparental caregiving could be regarded as a means of social engagement and social activity (Waldrop and Weber, 2001; Nyland et al., 2009; Arpino and Bordone, 2014; Burn and Szoeke, 2015; Bulanda and Jendrek, 2016), which could fulfill the need for social connectedness. As argued by disengagement theory, people gradually disengage from social life as they grow older (Cumming et al., 1960), making them more likely to lose social ties that were previously available to them. In this sense, grandparental caregiving becomes an important alternative because caring for children can be socially stimulating to the care provider. For example, in-depth interviews with 54 Caucasian grandparents conducted by Waldrop and colleagues reported that 11% of the grandparents perceived their lifestyle had become more active, and they enjoyed participating in activities with their grandchildren, such as team sports, school involvement, and meeting new people through their grandchildren’s activities (Waldrop and Weber, 2001). Similarly, research using survey data on early childhood care and development in China found that, for children who were enrolled in a preschool service, grandparents were more proactive in supporting language- and literacy-related events, including book reading, storytelling, singing, and family outings (Nyland et al., 2009). Another study, which examined the relationship between grandparenting roles and formal volunteering using data from the 2004 wave of the Health and Retirement Study, indicated that grandparents who provide non-residential care for grandchildren are more likely to engage in formal volunteering than grandparents not providing grandchild care (Bulanda and Jendrek, 2016). Moreover, grandparental caregiving may also increase daily activity, particularly for adults who may not otherwise be socially active. In one qualitative study aiming at investigating the effects of grandparental caregiving on American grandparents, Jendrek (1993) found that, among grandmothers who were providing regular daycare for their grandchildren, 35.4% of them claimed that their reason for caring for their grandchildren was that grandparental caregiving provided them with necessary everyday activities.

## Grandparental Caregiving, Reduced Lonely Dissatisfaction, and the Benefits

Loneliness is one major cause of life dissatisfaction, especially for older adults (Alpass and Neville, 2003; Luanaigh and Lawlor, 2008; Coyle and Dugan, 2012; Luo and Waite, 2014). Indeed, numerous studies have demonstrated that close social relations can provide a buffer against stress or anxiety (Bolger and Eckenrode, 1991; Schwarzer et al., 2014), bolster self-esteem (Hoffman et al., 1988), and inhibit depression (Tomita and Burns, 2013; also see a recent review, Cohen-Mansfield et al., 2016). Lonely dissatisfaction is defined as the older person’s acceptance or satisfaction with the amount of social interaction they are presently experiencing (Lawton, 1972, 1975).

It is suggested that social engagement in grandparental caregiving could increase intergenerational exchanges between grandparents and grandchildren (Bengtson, 2001; Uhlenberg, 2009), which could reduce dissatisfaction caused by loneliness to a larger extent. In China, grandparental caregiving is also a way to enjoy family support and maintain emotional closeness with family members, which, in turn, could lower the level of loneliness for grandparents (Goh, 2009). A growing body of empirical studies on empty-nest older adults suggests that, in comparison with the not empty nest group, the empty nest group has a higher level of loneliness, more health problems, and worse quality of life (Liu and Guo, 2007; Su et al., 2012; Wang et al., 2017). Another line of research offered more direct evidence showing that, in contrast to empty nest older adults, grandparents living in three-generational households – even in skipped-generation households – had better psychological well-being (Silverstein et al., 2006). Similarly, in one study conducted in Taiwan, researchers found that participants not providing grandchild care had a greater risk of feeling lonely and being depressed even after controlling for other potential confounding variables (Tsai et al., 2013).

Hence, in the present study, we also intend to examine the mediating role of lonely dissatisfaction in the association between grandparental caregiving and physical and mental health.

## The Present Study

In summary, grandparental caregiving as a form of social activity could promote social engagement and strengthen social connection in older adults, such that grandparents have claimed that playing with and caring for their grandchildren provides them with a form of daily activity and exercise, helping them to feel more socially engaged and less prone to lonely dissatisfaction. Hence, we would first expect an improvement in physical and mental health for grandparents who provide care to their grandchildren compared with those who do not provide any care (Hypothesis 1). As aforementioned, lonely dissatisfaction is known to influence many aspects of physical and mental health, and it may contribute to the relationship between grandparental caregiving and reported benefits. Thus, we further hypothesized that such a beneficial effect might be accounted for by reduced lonely dissatisfaction; in other words, lonely dissatisfaction might be a potential mediator between grandparental caregiving and physical and mental health (Hypothesis 2).

## Study 1: The Benefits of Grandparental Caregiving

In the first study, we aimed to investigate whether there were any differences in terms of physical and mental health between older adults who provide grandparental care and those who do not and whether these differences might be mediated by lonely dissatisfaction level. Moreover, as there is one possibility that cognitive abilities might be an important prior for grandparental caregiving (i.e., only older adults with sufficient cognitive resources would be more likely to engage in grandparental caregiving); hence, we also measured older adults’ cognition as a control variable in the present study.

## Materials and Methods

### Participants

Three hundred older adults aged 55–93 in Guangdong Province, China, were recruited using a stratified sampling method to participate in our survey. Two participants were excluded from the following analysis due to the incomplete responses. Eventually, a total of 298 older adults (aged 55–93, *M* = 62.99 years, *SD* = 5.92, 61% females) were included in the final analysis, and 148 of them (*N* = 148) were grandparents who have moved from a third-tier city to Guangzhou, a first-tier city, to provide grandparental care for their grandchildren (aged 55–77, *M* = 62.43 years, *SD* = 5.16, 61% females), and they have stayed in the large city for 73 months on average. The rest (*N* = 150) were grandparents who stayed in their original place, a third-tier city, without providing any grandparental care for their grandchildren (aged 55–93, *M* = 63.54 years, *SD* = 6.55, 61% females). All the participants were screened with the Mini-Mental State Examination (MMSE, Folstein et al., 1975), and they all earned MMSE scores greater than 27, which indicated that they had no cognitive impairment (Folstein et al., 1975). The survey was conducted with ethical approvals from IRB.

### Measures and Procedure

The study was conducted via paper and pen. All participants were asked to read and sign the consent form before answering any measures of psychological and cognitive functions. It took about 1 h for each participant to complete the questionnaire.

#### Demographic Information

Demographic information included age, sex (0 = female and 1 = male), educational level (0 = below primary; 1 = primary; 2 = junior high school; 3 = bachelor degree; 4 = master and above), self-reported health (from 1 = very poor to 5 = excellent). Self-reported SES was measured with the MacArthur Scale of Subjective Social Status, a pictorial representation that used a symbolic ladder to capture the subjects’ general social status based on usual socioeconomic status (SES) indicators (from 1 = lowest SES to 10 = highest SES; Adler and Stewart, 2007). These variables later served as control variables in the analysis.

#### Lonely Dissatisfaction

The Lonely Dissatisfaction Subscale of the Chinese version of the 23-item Philadelphia Geriatric Center Morale Scale (PGCMS, Lawton, 1972, 1975; Dong, 1999) was used to measure participants’ lonely dissatisfaction level. The subscale consisted of eight items, and participants only needed to answer “yes” or “no” for each item (refer to the Appendix for details of each item). In the present study, each high-morale response received a score of 0, and each low-morale response received a score of 1. Hence, the total scores ranged from 0 to 8, and larger scores indicated a higher level of lonely dissatisfaction for older adults. PGCMS consisted of three factors: agitation, attitude toward own aging, and lonely dissatisfaction (PGCMS, Lawton, 1972, 1975; Dong, 1999), which has been widely used to measure older adults’ morale or subjective well-being in aging research (Liang et al., 1987; Wong et al., 2004; Deng et al., 2010). Yao et al. (1995) used PGCMS to investigate the subjective well-being of older adults in Beijing, China, which indicated good test–retest reliability. In the present study, the subscale yielded acceptable internal consistency as indicated by Cronbach’s α = 0.63.

#### Physical and Mental Health

The Chinese version of the MOS 36-Item Short-Form Health Survey (SF-36) was used to assess participants’ physical and mental health (Ware et al., 1993, 1995; Li et al., 2003). The survey consisted of eight domains of health: physical functioning (PF), role limitations due to physical problems (RP), bodily pain (BP), general health (GH), vitality (VT), social functioning (SF), role limitations due to emotional problems (RE), and mental health (MH). They could be clustered into two component scores, namely the physical component summary (PCS) and mental component summary (MCS). Specifically, the PCS consisted of PF, RP, BP, and GH, and the MCS was composed of the other four domains. In the present studies, two component scores were computed following a three-step procedure developed by Ware et al. (1993). First, all eight original subscale scores were linearly transformed into eight scale scores ranging from 0 to 100. Second, a linear *z*-score transformation was performed to transform eight scores into eight *z*-scores, and then we averaged subscale *z*-scores to get two component *z*-scores. Finally, we obtained two component *t*-scores using linear *t*-score transformation (with mean 50 and standard deviation 10). A larger score indicated better health status.

#### Cognitive Functions

Cognitive functions of older adults were assessed by subtests of the Wechsler Adult Intelligence Scale-Revised Chinese Version (WAIS-RC) (Gong, 1992; Wechsler, 1997a, b), such as digit span-forward and backward tests and the digit symbol subtraction test, aiming at testing working memory and processing speed. In addition, a semantic word fluency test, asking participants to name as many animals as possible within 60 s (Wang et al., 2002), was also used to test participants’ vocabulary.

After finishing all measures, participants were thanked, and monetary incentives were given.

## Results and Discussion

### Descriptive Analysis

Table 1 reports participants’ demographic information in different grandparental caregiving groups. An independent sample *t*-test was conducted; no significant differences were found for all demographic variables (*t*s < 1.96) except for the self-reported SES, *t*(296) = 2.05, *p* = 0.04, Cohen’s *d* = 0.24, which indicated that grandparents who moved to the city to provide grandparental care had higher self-reported SES than those without providing grandparental care.

**TABLE 1:** Participant characteristics in grandparental and non-grandparental caregiving groups.

Measure	Caregiving group (*N* = 148)	Non-caregiving group (*N* = 150)	t/χ ^2^
		
	*M* (*SD*)	*M* (*SD*)	
Age	62.43 (5.16)	63.54 (6.55)	1.63
Gender	39% male	39% male	0.001
Education	1.67 (0.68)	1.78 (0.64)	1.44
Self-reported health	2.84 (0.80)	2.95 (0.87)	1.13
Self-reported SES	3.80 (1.62)	3.46 (1.25)	2.05*
Time stayed in city (month)	73.00 (55.16)	–	–
PGC-lonely dissatisfaction	2.03 (1.89)	2.50 (1.83)	2.20*
SF-36: PCS	51.59 (7.32)	48.57 (8.37)	3.32**
SF-36: MCS	50.93 (8.14)	49.35 (8.25)	1.66
Digit span-forward	7.16 (1.41)	6.63 (1.35)	3.35**
Digit span-backward	4.03 (1.17)	3.89 (1.14)	1.00
Digit symbol subtraction	23.57 (7.43)	19.77 (6.25)	4.78**
Word fluency	12.68 (4.36)	11.38 (4.17)	2.64**

*p < 0.05, **p < 0.01.

### Hypothesis Testing – Benefits of Grandparental Caregiving

Independent sample *t*-tests were conducted on lonely dissatisfaction and physical and mental health as well as cognitive functions between older adults who provided grandparental care and those who did not. Significant group differences were found on the lonely dissatisfaction, *t*(296) = 2.20, *p* = 0.03, Cohen’s *d* = 0.26; SF-36: PCS, *t*(296) = 3.32, *p* < 0.01, Cohen’s *d* = 0.39; digital span-forward, *t*(296) = 3.35, *p* < 0.01, Cohen’s *d* = 0.39; digit symbol subtraction test, *t*(296) = 4.78, *p* < 0.01, Cohen’s *d* = 0.55; and word fluency, *t*(296) = 2.64, *p* < 0.01, Cohen’s *d* = 0.31, whereas the other indicators were not significantly different between the two groups, *t*s < 1.66. The significant results were consistent with our first hypothesis, suggesting that older adults from the grandparental caregiving group have reduced lonely dissatisfaction and better physical health as well as cognition.

### Mediation Role of Lonely Dissatisfaction

**FIGURE 1:**
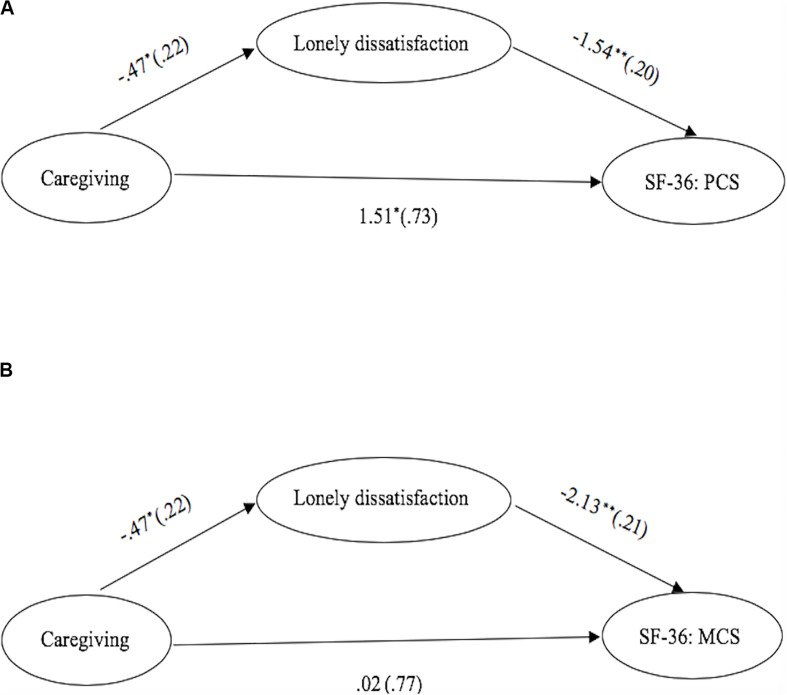
The Mediation of Physical and Mental Health by Lonely Dissatisfaction. **(A)** The mediation of physical health by lonely dissatisfaction scores for scores of SF-36: PCS. **(B)** The mediation of mental health by lonely dissatisfaction scores for scores of SF-36: MCS. Numbers are b-coefficients with the standard errors shown in parentheses. The values shown beneath the paths from caregiving to SF-36: PCS and SF-36: MCS represent the coefficients after the lonely dissatisfaction scores were added to the model. *p < 0.05, **p < 0.01.

Based on the results from independent samples *t*-tests, we further tested the mediation effect of lonely dissatisfaction on the association between grandparental caregiving status (dichotomous coding, 0 = no, 1 = yes) and outcome variables including SF-36: PCS and SF-36: MCS. The mediation models were tested with M*plus* 8 (Muthén and Muthén, 1998-2017) using the bootstrapping approach; 95% credibility intervals (CIs), not covering zero, indicated that the mediation effect of lonely dissatisfaction was significant. Age, sex, education level, self-reported health, SES, and four cognitive measures were included as covariates. The results indicated that, after controlling for demographic variables and cognitive functions, lonely dissatisfaction was a significant mediator between grandparental caregiving status and SF-36:PCS [*Estimate* = 0.73, *SE* = 0.35, 95% CI (0.057, 1.442)] as well as SF-36: MCS [*Estimate* = 1.01, *SE* = 0.47, 95% CI (0.083, 1.938)], supporting our second hypothesis (please refer to Table 2 and Figure 1 for detailed statistics), suggesting that compared with non-grandparental caregiving, the beneficial effect of grandparental caregiving might arise from reduced lonely dissatisfaction such that participants in the grandparent caregiving group had lower lonely dissatisfaction levels, which, in turn, could improve their physical and mental health. However, the current findings should also be interpreted with caution because the present study was cross-sectional in nature, and no causal inference could be made. One alternative might be that better physical and mental health made older adults more willing to provide grandparental care to their grandchildren rather than vice versa. Therefore, we further conducted a small follow-up in a 6-month interval to validate our findings.

**TABLE 2:** Mediating effect of lonely dissatisfaction in Study 1 (*N* = 298).

Parameter	Mediation model		Parameter	Mediation model	
	Estimate (SE)	95% CI		Estimate (SE)	95% CI
SF-36: PCS ←			SF-36: MCS ←		
Age	−0.32*(0.07)	[−0.45, −0.17]	Age	−0.16* (0.07)	[−0.31, −0.02]
Gender	0.48 (0.73)	[−0.98, 1.86]	Gender	−0.09 (0.76)	[−1.61, 1.39]
Education	0.64 (0.64)	[−0.57, 1.95]	Education	1.03 (0.62)	[−0.16, 2.27]
Self-reported health	1.85*(0.46)	[0.94, 2.73]	Self-reported health	0.97* (0.48)	[0.03, 1.91]
Self-reported SES	0.31 (0.29)	[−0.23, 0.92]	Self-reported SES	0.09 (0.31)	[−0.48, 0.72]
Digit span-forward	0.88* (0.31)	[0.27, 1.50]	Digit span-forward	1.13* (0.33)	[0.50, 1.80]
Digit span-backward	0.11 (0.38)	[−0.64, 0.85]	Digit span-backward	0.28 (0.42)	[−0.54, 1.10]
Digit symbol subtraction	0.01 (0.06)	[−0.11, 0.14]	Digit symbol subtraction	−0.07 (0.06)	[−0.19, 0.06]
Word fluency	0.07 (0.09)	[−0.13, 0.23]	Word fluency	0.14 (0.10)	[−0.06, 0.32]
Caregiving	1.51* (0.73)	[0.10, 2.93]	Caregiving	0.02 (0.77)	[−1.51, 1.49]
Lonely dissatisfaction	−1.54* (0.20)	[−1.92, −1.14]	Lonely dissatisfaction	−2.13* (0.21)	[−2.53, −1.70]
Lonely dissatisfaction ←			Lonely dissatisfaction ←		
Caregiving	−0.47* (0.22)	[−0.89, −0.04]	Caregiving	−0.47* (0.22)	[−0.89, −0.04]

*CI does not cover zero indicating significance.

### Study 2: A Longitudinal Follow-Up

In the follow-up, the same measures as Study 1 were used to compare the within-individual changes with time in terms of physical and mental health among participants from different grandparental caregiving groups, aiming at a better understanding of the causal relations between grandparental caregiving, lonely dissatisfaction, and outcomes.

## Materials and Methods

### Participants and Procedure

The longitudinal follow-up was conducted 6 months after Study 1. Participants were randomly selected from Study 1. Excel 2016 was used to generate random integers that didn’t repeat, corresponding to the selected participant ID in Study 1. Fifty-two older adults (aged 55–76, *M* = 62.89 years, *SD* = 5.32, 58% females) who have provided grandparental care and 50 older adults (aged 55–93, *M* = 64.56 years, *SD* = 7.14, 54% females) who stayed in their original residence without providing any grandparental care were randomly recruited and interviewed^1^. The same measurements including demographics, Lonely Dissatisfaction Subscale of PGCMS, and SF-36 as well as cognitive functions were administrated. All measures yielded good internal consistencies (α > 0.67) as well as test–retest reliability (*r* > 0.72).

### Analytical Strategy

First, a mixed-model ANOVA was conducted on different outcome variables with time (T1 vs. T2) as the within-subject factor and grandparental caregiving group (yes vs. no) as the between-subject factor to examine whether the time-related changes in physical and mental health were different between the two groups. Next, mediation analysis would be conducted to further test the role lonely dissatisfaction played in affecting outcomes. Because this study was a longitudinal follow-up, in the mediation analysis, residuals of lonely dissatisfaction (i.e., Δ lonely dissatisfaction) and other outcome variables instead of the raw scores were used as a mediator and DVs, which were obtained by using scores at T2 as dependent variables and scores at T1 as predictors in regression models following the residual change score method (Traub, 1967; Rowan et al., 2017). Positive residuals indicated an increase, and negative residuals indicated a reduction in the corresponding variable over time.

## Results and Discussion

### Descriptive Statistics

Table 3 described the basic demographic information of the two grandparental caregiving groups. An independent sample *t*-test indicated that, similar to Study 1, there was only a marginally significant difference in self-reported SES between the two groups, *t*(100) = 1.85, *p* = 0.067, Cohen’s *d* = 0.37. The rest, such as age, sex, and self-reported health was not significantly different between groups (*t*s < 1.35).

**TABLE 3:** Participants characteristics in two grandparental caregiving groups.

Measure	Caregiving group (*N* = 52)	Non-caregiving group (*N* = 50)	
		
	*M* (*SD*)	*M* (*SD*)	t/χ ^2^
Age	62.89 (5.32)	64.56 (7.14)	1.35
Gender	42% of men	46% of men	0.14
Education	1.63 (0.56)	1.66 (0.72)	0.20
Self-reported health	2.90 (0.91)	2.94 (0.87)	0.21
Self-reported SES	4.06 (1.50)	3.54 (1.31)	1.85^+^
Duration of been in city (month)	68.58 (54.73)	–	–

*^+^Denotes marginally significance.*

### Longitudinal Changes

**FIGURE 2:**
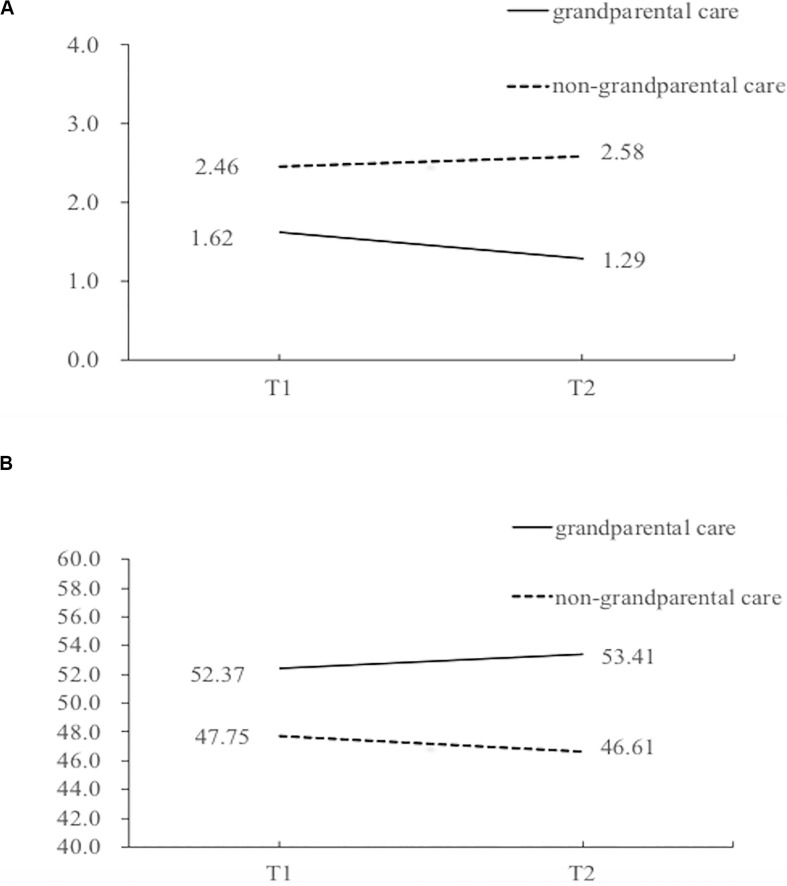
Changes of Lonely Dissatisfaction and SF-36: MCS across Time in Different Groups. **(A)** Level of Lonely Dissatisfaction across Time. **(B)** Level of SF-36: MCS across Time.

A mixed-model ANOVA found a significant Time x Group interaction for SF-36: MCS, digital span-backward, digit symbol subtraction test and word fluency, *F*(1, 100) = 4.12, *p* = 0.045, partial η^2^ = 0.04; *F*(1, 100) = 11.62, *p* < 0.001, partial η^2^ = 0.10; *F*(1, 100) = 6.91, *p* = 0.01, partial η^2^ = 0.07; *F*(1, 100) = 6.02, *p* = 0.016, partial η^2^ = 0.06, respectively, although the time x group interaction for lonely dissatisfaction, SF-36: PCS and digital span-forward was not significant, *F*(1, 100) = 2.82, *p* = 0.096, partial η^2^ = 0.027; *F*(1, 100) = 1.29, *p* = 0.26, partial η^2^ = 0.013; and *F*(1, 100) = 0.55, *p* = 0.46, partial η^2^ = 0.006, respectively (please also refer to Table 4). These interactions suggest that older adults who provided grandparental caregiving had greater improvement in their lonely dissatisfaction and mental health in the 6-month interval (Figure 2).

**TABLE 4:** Lonely dissatisfaction, physical and mental health, as well as cognitive functions across time in two groups.

	Caregiving group (*N* = 52)		Non-caregiving group (*N* = 50)		F_*condition*_	F_*time*_	F_*interaction*_
Measure	T1	T2	T1	T2			
					
	*M* (*SD*)	*M* (*SD*)	*M* (*SD*)	*M* (*SD*)			
Lonely dissatisfaction	1.62 (1.68)	1.29 (1.47)	2.46 (1.76)	2.58 (1.96)	11.51**	0.60	2.82
SF-36: PCS	53.15 (6.80)	53.72 (6.80)	46.67 (8.04)	46.21 (8.45)	24.06**	0.01	1.29
SF-36: MCS	52.37 (7.33)	53.41 (7.58)	47.75 (8.65)	46.61 (8.99)	14.04**	0.00	4.12*
Digit span-forward	7.48 (1.34)	7.60 (1.29)	6.48 (1.25)	6.54 (1.27)	16.68**	5.55*	0.55
Digit span-backward	4.10 (1.32)	4.42 (1.35)	3.78 (1.33)	3.78 (1.31)	3.44	11.62**	11.62**
Digit symbol subtraction	25.64 (7.70)	26.96 (6.96)	20.22 (6.97)	19.90 (6.88)	20.45**	2.58	6.91**
Word fluency	13.50 (4.11)	15.40 (4.29)	11.70 (5.59)	12.48 (5.57)	6.21*	34.31**	6.02*

*p < 0.05, **p < 0.01.

### Mediation Role of Lonely Dissatisfaction

**FIGURE 3:**
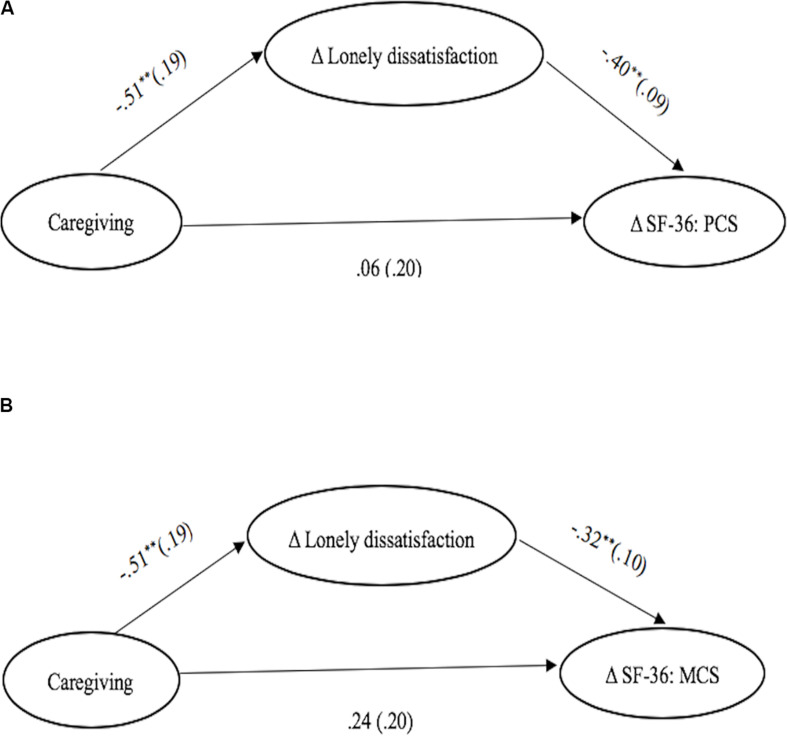
The Mediation of Δ SF-36: PCS and Δ SF-36: MCS by Δ Lonely Dissatisfaction across Time. **(A)** The mediation of changes of physical health by changes of lonely dissatisfaction scores for changes of scores of SF-36: PCS. **(B)** The mediation of changes of mental health by changes of lonely dissatisfaction scores for changes of scores of SF-36: MCS. Numbers are b-coefficients with the standard errors shown in parentheses. The values shown beneath the paths from caregiving to changes of SF-36: PCS and SF-36: MCS represent the coefficients after the changes of lonely dissatisfaction scores were added to the model. *p < 0.05, **p < 0.01.

Identical mediation analysis based on the bootstrapping approach with M*plus* 8 (Muthén and Muthén, 1998-2017) as Study 1 used with the exception that residual scores (between T1 and T2) of lonely dissatisfaction and outcomes were used as mediator and dependent variables. The results represented in Table 5 demonstrate that the effect of grandparental caregiving on changes of physical and mental health within a 6-month interval were all mediated by changes in lonely dissatisfaction, that is, for Δ SF-36: PCS [*Estimate* = 0.21, *SE* = 0.09, 95% CI (0.05, 0.39)], and for Δ SF-36: MCS [*Estimate* = 0.17, *SE* = 0.09, 95% CI (0.03, 0.37)] even when controlling for age, sex, education level, self-reported health, and SES reported at T1 as well as changes of cognitive functions within 6 months (Figure 3). Such findings further support our second hypothesis, suggesting that grandparental caregiving made older adults more likely to report reduced feelings of lonely dissatisfaction, and such reduction in lonely dissatisfaction could contribute to improvements in physical and mental health.

**TABLE 5:** Mediation effect of Δ lonely dissatisfaction (*N* = 102).

Parameter	Mediation model		Parameter	Mediation model	
	Estimate (SE)	95% CI		Estimate (SE)	95% CI
Δ SF-36: PCS ←			Δ SF-36: MCS ←		
Age	−0.03 (0.02)	[−0.06, 0.01]	Age	−0.02 (0.02)	[−0.05, 0.01]
Gender	−0.10 (0.18)	[−0.44, 0.26]	Gender	−0.02 (0.20)	[−0.43, 0.36]
Education	0.03 (0.14)	[−0.27, 0.29]	Education	0.01 (0.14)	[−0.29, 0.28]
Self-reported health	−0.05 (0.12)	[−0.29, 0.18]	Self-reported health	0.00 (0.12)	[−0.24, 0.23]
Self-reported SES	−0.05 (0.06)	[−0.17, 0.08]	Self-reported SES	−0.01 (0.06)	[−0.13, 0.12]
Δ Digit span-forward	0.15 (0.09)	[−0.04, 0.32]	Δ Digit span-forward	0.13 (0.09)	[−0.05, 0.29]
Δ Digit span-backward	0.02 (0.13)	[−0.22, 0.27]	Δ Digit span-backward	−0.00 (0.14)	[−0.27, 0.27]
Δ Digit symbol subtraction	−0.20* (0.09)	[−0.38, −0.03]	Δ Digit symbol subtraction	−0.14 (0.10)	[−0.34, 0.05]
Δ Word fluency	0.33* (0.12)	[0.10, 0.59]	Δ Word fluency	0.28* (0.14)	[0.00, 0.56]
Caregiving	0.06 (0.20)	[−0.35, 0.42]	Caregiving	0.24 (0.20)	[−0.17, 0.60]
Δ Lonely dissatisfaction	−0.40* (0.09)	[−0.58, −0.21]	Δ Lonely dissatisfaction	−0.32* (0.10)	[−0.52, −0.15]
Δ Lonely dissatisfaction ←			Δ Lonely dissatisfaction ←		
Caregiving	−0.51* (0.19)	[−0.89, −0.14]	Caregiving	−0.51* (0.19)	[−0.89, −0.14]

^Δ^ The residues of variables from T1 to T2. *CI does not cover zero indicating significance.

## General Discussion

Data from both cross-sectional and short-term longitudinal studies confirm our original hypotheses, supporting that providing grandparental care could benefit older adults to a larger extent compared with those who did not. And such beneficial effect was reached through reduced feelings of lonely dissatisfaction in older adults. Theoretically, grandparental care could have both beneficial and detrimental effects on older adults’ health. For example, a stress process model suggests that negative health effects may develop from the overload and strain associated with older adults’ roles as caregivers for their grandchild (Pearlin et al., 1989). Role enhancement theory alternatively argues that older adults’ health may be improved by an accumulation of multiple roles due to increased social support from their social roles (Szinovacz and Davey, 2006).

The majority of studies on the grandparent–grandchild relationship and its effect on older adults have been focused on grandparents’ responsibilities for the care of grandchildren (i.e., custodial grandparental caregiving) and reported the possible risks on their physical health and depressive symptoms (Blustein et al., 2004; Hayslip and Kaminski, 2005; Hughes et al., 2007; Baker and Silverstein, 2008; Grundy et al., 2012; Hayslip et al., 2014). However, another line of research on migrant grandparental caregiving has suggested the opposite. For example, King and colleagues found that migration of the older generation didn’t necessarily lead to adverse effects because living with adult children would enable them to get access to advanced health care more easily (King et al., 2014). In addition, research regarding migrant/immigrant Chinese grandparenting suggests that, despite migration, Chinese grandparents consider their caregiving experiences positive, and most grandparents choose to focus on certain aspects of successful grandparenting experiences (Xie and Xia, 2011; Chen and Lewis, 2015; Zhu et al., 2019). Our findings from both cross-sectional and longitudinal designs further confirm the physical and mental benefits of migrant grandparents.

In the present study, we tested our hypotheses in the context of rapid urbanization in China, which has revealed the beneficial effects of grandparental caregiving. One possibility is that the Chinese culture (or collectivistic cultures in general) might emphasize interpersonal harmony, familism, and intergenerational transfers to a larger extent (Chen et al., 2011), and making grandparental caregiving an important alternative to reach intergenerational harmony and fulfill the responsibility of intergenerational transfers through grandparent–grandchild intergenerational exchange could eventually promote older adults’ mental health (Perissinotto et al., 2012). The generalizability of the present findings might be unclear. However, we still believe that such results might be true in Western cultures as well, especially seeing the ascending rates of grandparental caregiving in Western countries, such as Europe and the United States (Guzman, 2004; Hank and Buber, 2009). Some previous studies might provide some indirect support. For example, studies in the United States using a representative longitudinal data set found similar benefits to grandmothers who babysit (Hughes et al., 2007). A recent study conducted in 10 European countries also found that providing up to 15 h a week of childcare helps maintain grandparents’ health and well-being (Glaser et al., 2014).

## The Role of Lonely Dissatisfaction in Grandparental Caregiving

Previous longitudinal studies have offered some related evidence for the long-term beneficial effect of grandparental caregiving. For example, using data from the Survey of Health, Aging and Retirement in Europe (SHARE), Di Gessa et al. (2015) found that both intensive and non-intensive grandchildren care could enhance older adults’ physical health. However, there is one question that remains open in previous studies, i.e., the underlying mechanism behind the gains caused by grandparental caregiving. Our results provided some preliminary insights, suggesting that reduced lonely dissatisfaction associated with grandparental caregiving (Bengtson, 2001; Uhlenberg, 2009) might be one of the reasons. Much of the existing research has demonstrated that loneliness and life dissatisfaction could have dramatic negative effect on older adults’ physical and mental health (Coyle and Dugan, 2012; Guven and Saloumidis, 2014; Marum et al., 2014; Hülür et al., 2017). Such interactions with children as those brought about by grandparental caregiving can allow older adults to maintain an active lifestyle and strengthen intergenerational ties with grandchildren, which, in turn, results in enhanced physical and mental health (Balukonis et al., 2008; Chen et al., 2011).

## Grandparental Caregiving and Cognitive Functions

Another interesting finding is also worth noticing: An improvement in cognitive functions (e.g., digit span-backward, digit symbol subtraction test, and semantic word fluency) was observed at the 6-month interval for those who provided grandparental care. These findings offer some evidence to the literature on grandparental caregiving and cognitive functioning among older adults. Indeed, several studies show that grandparental caregiving could enhance cognitive functioning of grandparents. For example, Arpino and Bordone (2014) found that providing childcare had a substantial and positive effect on the enhancement in grandmothers’ verbal fluency independent of the frequency of grandparental caregiving using the instrumental variable approach to address the endogeneity problem based on the data from SHARE (Health, Aging and Retirement in Europe), which was later confirmed by Ahn and Choi (2019) and Sneed and Schulz (2019) with other archived data. One speculation would be that reduced lonely dissatisfaction could also work for such enhancement in cognition. For example, there has been longitudinal evidence suggesting that loneliness could also influence older adults’ cognitive functions (Zhong et al., 2016; Donovan et al., 2017; Rafnsson et al., 2017).

## Limitations and Future Directions

Several limitations of the present study should be noted. First, the benefits of grandparental caregiving we found might have a self-selecting bias such that in the follow-up study, we observed that older adults who provided grandparental care exhibited higher scores in almost all outcome measures at baseline (see Table 4 and Figure 2), which might suggest that those older adults who felt in good health (both physically and mentally) were more willing to provide grandparental care (Arpino and Bordone, 2014), and such self-selection could have an accumulative effect on their physical and mental health compared with those who did not provide any grandparental care. Future studies could also test if older adults with poor physical and mental health could gain similar benefits by providing grandparental care.

Second, although we conducted the follow-up study, we still noted that the follow-up period might be a little bit too short to detect significant changes in physical and mental health. However, our results suggest that the changes were evident at the 6-month period. Future studies could further test the changes in grandparents’ physical and mental health at a longer period of time so as to capture more significant and robust effects. Third, feelings of lonely dissatisfaction are associated with marital status and living arrangement of older adults such that those who are living alone or widowed have a greater risk of experiencing loneliness and dissatisfaction (De Jong Gierveld et al., 2012; Vignoli et al., 2014). Whereas our studies have not taken these potential confounding factors into account, they could be included in future research. Finally, the grandparental caregiving group and the non-caregiving group differed in migration status, and there might be confounding effects of migration with grandparental caregiving. Hence, further studies should be conducted to clearly distinguish the respective effect of migration and grandparental caregiving on grandparents via including another two groups: a group of participants performing grandchildren care in their original place, and another group of participants who move to cities without providing grandchildren care.

Despite these limitations, the present study contributes to the debate on the effects of grandparental caregiving on older adults’ physical and mental health, suggesting that grandparental caregiving is indeed beneficial for older adults (Hughes et al., 2007; Powdthavee, 2011) and further demonstrates that such benefits could be reached through reduced lonely dissatisfaction.

## Data Availability Statement

The datasets generated for this study are available on request to the corresponding author.

## Ethics Statement

The studies involving human participants were reviewed and approved by the Ethics Committee of School of Psychological and Cognitive Sciences, Peking University. The patients/participants provided their written informed consent to participate in this study.

## Author Contributions

YC and XZ contributed to the study design, data analysis, interpretation of results, and writing of the manuscript. YL contributed to the recruitment of participants and writing of the manuscript. All authors have approved the final manuscript.

## Conflict of Interest

The authors declare that the research was conducted in the absence of any commercial or financial relationships that could be construed as a potential conflict of interest.

## Footnotes

1 We tested the differences, in terms of lonely dissatisfaction and physical and mental health as well as cognitive functions between those who were selected and who weren’t selected for caregiving group and non-caregiving group with a general linear model (GLM multivariate model). The results show that only physical health and the digit symbol subtraction test were found to exhibit significant differences, *F*(1, 287) = 5.76, *p* < 0.05, partial η*2* = 0.02 and *F*(1, 287) = 6.52, *p* < 0.05, partial η^2^ = 0.02.

## Appendix

**TABLE A1:** Lonely dissatisfaction subscale of the Philadelphia geriatric center morale scale.

Item	Yes	No
I feel lonely most of the time.	Yes	No
I see enough of my friends and relatives.	Yes	No
I sometimes feel that life is not worth living.	Yes	No
Life is hard for me most of the time.	Yes	No
I am satisfied with my life today.	Yes	No
I have a lot to be sad about.	Yes	No
People had it better in the old days.	Yes	No
A person has to live for today and not worry about tomorrow.	Yes	No

**TABLE A2:** Physical component score (PCS) and mental component score (MCS) (Study 1).

	Caregiving group (*N* = 148)	Non-caregiving group (*N* = 150)
	
Measures	*M* (*SD*)	*M* (*SD*)
**PCS**	51.59 (7.32)	48.57 (8.37)
PF	80.14 (17.28)	70.13 (23.86)
RP	69.68 (19.92)	67.29 (21.56)
BP	73.36 (18.30)	69.15 (18.10)
GH	58.54 (18.47)	50.75 (18.74)
**MCS**	50.93 (8.14)	49.35 (8.25)
VT	64.74 (19.72)	62.17 (18.57)
SF	72.30 (20.13)	67.08 (19.56)
RE	71.06 (20.28)	68.28 (22.64)
MH	71.89 (17.01)	69.93 (17.14)

**TABLE A3:** Physical component score (PCS) and mental component score (MCS) (Study 2).

	Caregiving group (*N* = 52)	Non-caregiving group (*N* = 50)
	
Measures	*M* (*SD*)	*M* (*SD*)
**PCS**	53.72 (6.80)	46.21 (8.45)
PF	86.44 (16.01)	72.30 (22.04)
RP	84.62 (15.78)	69.13 (23.06)
BP	82.79 (17.21)	67.36 (19.05)
GH	58.54 (18.47)	50.75 (18.74)
**MCS**	53.41 (7.58)	46.61 (8.99)
VT	69.83 (18.64)	58.75 (17.90)
SF	85.58 (17.04)	67.25 (20.81)
RE	81.41 (18.86)	60.00 (23.93)
MH	76.54 (16.10)	63.80 (18.06)
